# Prediction of Treatment Week Eight Response & Sustained Virologic Response in Patients Treated with Boceprevir Plus Peginterferon Alfa and Ribavirin

**DOI:** 10.1371/journal.pone.0103370

**Published:** 2014-08-01

**Authors:** Alex Thompson, Scott Devine, Mike Kattan, Andrew Muir

**Affiliations:** 1 Department of Gastroenterology, St. Vincent's Hospital, Melbourne, VIC, Australia; 2 Merck, Whitehouse Station, New Jersey, United States of America; 3 Department of Quantitative Health Sciences, Cleveland Clinic, Cleveland, Ohio, United States of America; 4 Duke University School of Medicine, Durham, North Carolina, United States of America; University of Sydney, Australia

## Abstract

**Aim:**

Sustained virologic response (SVR) can be attained with boceprevir plus peginterferon alfa and ribavirin (PR) in up to 68% of patients, and short duration therapy is possible if plasma HCV RNA levels are undetectable at treatment week 8 (TW8 response). We have developed predictive models for SVR, and TW8 response using data from boceprevir clinical trials.

**Methods:**

Regression models were built to predict TW8 response and SVR. Separate models were built for TW8 and SVR using baseline variables only, and compared to models with baseline variables plus HCV RNA change after 4 weeks of PR (TW4 delta). Predictive accuracy was assessed by c-statistics, calibration curves, and decision curve analyses. Nomograms were developed to create clinical decision support tools. Models were externally validated using independent data.

**Results:**

The models that included TW4 delta produced the best discrimination ability. The predictive factors for TW8 response (n = 856) were TW4 delta, race, platelet count and ALT. The predictive factors for SVR (n = 522) were TW4 delta, HCV-subtype, gender, BMI, RBV dose and platelet count. The discrimination abilities of these models were excellent (C-statistics = 0.88, 0.80 respectively). Baseline models for TW8 response (n = 444) and SVR (n = 197) had weaker discrimination ability (C-statistic = 0.76, 0.69). External validation confirmed the predictive accuracy of the week 4 models.

**Conclusions:**

Models incorporating baseline and treatment week 4 data provide excellent prediction of TW8 response and SVR, and support the clinical utility of the lead-in phase of PR. The nomograms are suitable for point-of-care use to inform individual patient and physician decision-making.

## Introduction

Chronic infection with hepatitis C virus (HCV) affects approximately 130–170 million individuals worldwide.[1] It is associated with the development of liver failure and hepatocellular carcinoma and is the leading indication for liver transplantation in developed countries. Morbidity and mortality may be prevented by antiviral therapy and viral clearance. Until 2011, the standard of care treatment for genotype 1 HCV was dual therapy with peginterferon-alfa and ribavirin (PR). Unfortunately the success rate was less than 50%, and treatment was frequently associated with significant toxicity. For this reason, much effort has been invested in the development of new treatment for HCV, leading to the recent approval of the first generation HCV protease inhibitors.[2]–[5]

Triple therapy with boceprevir plus PR is associated with rates of sustained virological response (SVR) of up to 68% in treatment-naïve patients. Boceprevir therapy also offers the possibility of shortened duration therapy for rapid responders, defined by an undetectable plasma HCV RNA level at treatment week 8 (TW8). Whilst boceprevir represents a very significant therapeutic advance, treatment is expensive, there remains the potential for side effects, and response rates vary depending on multiple underlying factors, including host and viral characteristics and interferon responsiveness.[6] In addition, the treatment landscape is rapidly shifting, with multiple other compounds now in phase 3 trials. For these reasons, treatment decisions continue to be individualized.

Clinical tools for predicting individualized probability of achieving SVR, as well as TW8 response, would be useful for informing patient and physician clinical decision-making about the success and potential duration of treatment. The baseline characteristics that have been associated with response to boceprevir-based therapy include host factors (IL28B genotype, liver fibrosis stage, ethnicity, body mass index (BMI), age and statin use),[4], [6] viral factors (HCV-1 subtype 1b vs. 1a, baseline HCV RNA level),[4], [6] and interferon responsiveness (past treatment response, as well as on-treatment viral decline during the first 4 weeks of lead-in PR therapy).[4], [6] Despite the recognition that these variables are important, predicting response for an individual patient remains difficult.

The aim of this analysis was therefore to develop simple clinical tools that could be used in clinical practice to guide treatment decisions by accurately predicting the likelihood of an individual i) achieving an SVR; and/or ii) being eligible for short duration therapy, with boceprevir-based triple therapy. We have developed two different models for predicting SVR as well as TW8 response using data from the SPRINT-2[4] (Clinicaltrials.gov Trial # NCT00705432), RESPOND-2[2] (Clinicaltrials.gov Trial # NCT00708500), and PROVIDE[7] (Clinicaltrials.gov Trial # NCT00910624) clinical trials. The models were then externally validated using data from a post-registration anemia management study (Clinicaltrials.gov Trial # NCT01023035).[8], [9] Simple nomograms designed for point-of-care clinical use were then developed. These data-driven tools will supplement clinical judgment on the choice of therapy and inform patient-physician decision-making on the appropriate initiation of treatment for chronic HCV infection.

## Methods

### Patient Population

We utilized data from boceprevir treated patients who participated in the SPRINT-2, RESPOND-2 and PROVIDE studies. While these studies had different designs and different treatment populations, the three studies captured each of the four critical subpopulations of clinical interest to a practicing clinician. The SPRINT-2 study enrolled treatment-naïve individuals who were chronically infected with genotype 1 HCV. All patients were ≥18 years of age, with a plasma HCV RNA level ≥10,000 IU per milliliter. The RESPOND-2 study enrolled patients who had previously failed a course of PR. Patients were either relapsers or partial responders to a prior course of PR, according to standard definitions. Prior null responders to PR were excluded, where non-response was defined as a decline in the HCV RNA level <2 log10 IU/mL from baseline after 12 weeks of PR. The PROVIDE study enrolled patients who were randomized to the PR control arm in one of the phase 2/3 studies of boceprevir (SPRINT-1,[10] SPRINT-2, RESPOND-2 and peginterferon-α2a/boceprevir study[11]), but did not achieve SVR. The PROVIDE study therefore included patients who were well-characterized relapsers, partial responders and null responders.

There were multiple patient types and corresponding regimens across the three trials used to build the model. The overarching goal of the patient selection approach was to develop models with data from patients that received treatment consistent with the boceprevir product label in the United States. Therefore, we did not consider patients who received treatment that was outside the label. For example, in the SPRINT-2 trial only patients who did not have cirrhosis were evaluated from the response-guided therapy (RGT) boceprevir trial arms(Arm 2). However, in patients with cirrhosis and prior null responders are not eligible for RGT and thus those patients from the RGT arm (Arm 2) were excluded from the sample used to develop the models of TW8 response due to their ineligibility for at shortened course of therapy. When developing the SVR only models, patients with cirrhosis and prior null responders were included in the sample from only the non-RGT arm (Arm 3) because this represented the recommended treatment course a clinician would be expected to use based upon the product labeling. In addition to the treatment regimen, there were a number of factors that determined a patient final inclusion from the sample used to develop either model. Patients who discontinued treatment early, either because of adverse events, or protocol violations, was not considered for the modeling exercise, as one would not expect them to achieve a TW8 nor a SVR because of their failure to complete therapy.

### Outcomes of Interest

We considered two clinically relevant endpoints for model building. The first model predicted SVR, which is the primary endpoint of treatment. SVR was defined as having undetectable HCV RNA levels 24 weeks after the completion of therapy. If HCV RNA measurements for this time point or later were missing, the 12-week post-treatment measurement was used. The second model was built to predict a response at treatment week eight (TW8). Response at TW8 is a critical time-point for the determination of overall length of therapy for patients eligible for RGT. Undetectable HCV RNA at TW8 defined TW8 response. Plasma HCV RNA levels were measured using the TaqMan 2.0 assay (Roche Diagnostics), which has a lower limit of quantification of 25 IU/mL and a lower limit of detection of 9.3 IU/mL. For all analyses, undetectable plasma HCV RNA was defined using the lower limit of detection (9.3 IU/mL). The modeling considered the modified intention-to-treat population, consisting of patients who completed the lead-in period of treatment and received at least one dose of boceprevir or placebo.[2], [4]

### Candidate Predictors

As this study was conducted with data collected from the boceprevir clinical trial program, potential predictors were limited to those that were collected at the time of the study. All variables from the SPRINT-2 and RESPOND-2 data sets used in the analysis were from the final clinical trial datasets. All variable definitions, categories, groupings and scales were maintained to ensure consistency with previously published information.[2], [4] Candidate predictors were selected based upon clinician judgment, variable availability within the clinical trial data and the theoretical relevance of the variable to the outcomes of interest. Predictors were further scrutinized for routine availability in clinical practice, relative expense and the invasiveness of the measure. Full models were developed for the TW8 and SVR data. These models included prior PR treatment experience type (untreated, partial responder, relapser or null responder), IL28B genotype (CC vs. non-CC for rs12979860), HCV genotype 1 (G1) subtype, ribavirin starting dose (mg), age, ethnicity (black vs. non-black), gender, baseline values for weight (kg), BMI (kg/m2), hemoglobin (g/dL), ALT to ULN ratio, platelet count, statin use, and plasma HCV RNA level, as well as plasma HCV RNA after 4 weeks of PR (TW4), log_10_ change in plasma HCV RNA from baseline after 4 weeks of PR (TW4 response). Although liver histology was available for this cohort, the decision was made not to include this variable in the models, given the general trend away from liver biopsy in clinical management.

### Prediction Model Development

For each clinical endpoint, we built two separate models – the first considering only baseline variables as predictors, the second included information on the log_10_ change in plasma HCV RNA from baseline after the four week PR only exposure (i.e. TW4 response). Interferon responsiveness, indicated by a rapid reduction in plasma HCV RNA during the four-week lead-in phase of PR treatment, has been shown to be a strong predictor of SVR.[12], [13] Logistic regression was used to develop both models. Linearity assumptions were relaxed with use of restricted cubic splines. Bootstrapping, with 1000 resamples, was used in conjunction with estimation of discrimination and calibration. Discrimination was measured with the concordance index,[14] and calibration was evaluated graphically by plotting predicted vs. observed proportions. Decision curve analysis was also performed.[15] Cross validation by dataset was used to verify insensitivity to dataset for both discrimination and calibration. To reduce the number of predictors needed by the end user, a step down approach was used.[16] The step down approach uses variable selection to approximate the full model by predicting its linear predictor. When missing values for predictor variables were identified, multiple imputation with chained equations (MICE) techniques were utilized.[17] This approach uses Gibbs sampling and a series of conditional models to arrive at imputations for missing values. We developed a nomogram for each model. A nomogram provides an individualized prediction tool for an outcome based upon the characteristics of a given patient. Nomograms are developed based upon the linear predictors generated from the regression models. A detailed description of the methods used in nomogram development can be found elsewhere. All analyses were performed using R (Version 2.14, R Foundation for Statistical Computing, Vienna, Austria, 2012) with *Hmisc*,[18] *rms* [19] and *mice* [17] packages.

### Decision Tree Development

As an alternative approach to the nomograms, recursive partitioning analyses were conducted. Recursive partitioning creates a decision tree that attempts to correctly classify members of a population based upon multiple dichotomous splits of independent variables. The hypothesis was that patient features could be examined to find groups of patients for whom the outcome could be predicted with similar accuracy compared to the nomograms risks from the nomograms. In effect this attempts to model the utility of “counting” recognized risk factors to predict the outcome for an individual. In the first two analyses, trees were constructed to find groups of patients with relatively homogeneous predicted probabilities for TW8 response and sustained virologic response. The outcome variable being examined by the recursive partitioning trees was the predicted probability of the outcomes from the nomograms, not the actual outcome. This process kept the underlying nomogram model intact while attempting to identify groups from the nomogram that have similar probabilities within a group of the outcomes of interest. Continuous variable cut points were determined to optimize categorization. The response variable was formed using the leave-one-out method to facilitate the calculation of overfit-bias reduced estimates. Box-and-whisker plots were produced representing the range of predicted probabilities within each group created by the recursive partitioning process.

### External Validation

Finally, we conducted an external validation of our nomogram using data from the management of anemia study (NCT01023035).[8], [9] This randomized, multi-center, parallel-arm, open-label trial of previously untreated non-cirrhotic patients compared the effect on efficacy of erythropoietin versus RBV dose reduction for the management of anemia in subjects who became anemic during triple therapy with boceprevir plus PR. Inclusion criteria differed from the registration studies by requiring a baseline hemoglobin (Hb) <15 g/dL. Subjects followed similar treatment regimens to those found in the product labeling. In the event of anemia (Hb <1 g/dL), patients were randomized to erythropoietin vs. RBV dose reduction alone. We used the *val.prob* R package to compare the predicted probabilities based upon the nomograms to the actual outcomes of TW8 undetectability and SVR found in the management of anemia study. [20] C-statistics and calibration assessments were determined for each model against the validation data set.

## Results

### Prediction Models

1,111 patients were evaluated from the SPRINT-2, RESPOND-2 and PROVIDE trials. Counts and variable availability for patients used in the TW8 and SVR model development appear in Table 1. Values where imputed for BMI and METAVIR score on one patient and statin use and initial ribavirin dose on 66 patients. IL28B genotype was only available for a subset of the SPRINT-2, RESPOND-2 cohorts, and not for the PROVIDE cohort, thus baseline models for TW8 (n = 444) and SVR (n = 197) were limited to previously untreated, relapsers and partial responders. The variables included in the TW8 model were age, race, METAVIR score, steatosis score, statin use, platelets, ALT to ULN ratio, IL28 genotype, and previous treatment experience and the variables included in the SVR model were race, steatosis score, statin use, ribavirin, platelets, IL28 genotype, and HCV G1 subtype. We were unable to fit prior relapsing patients in any model, thus forcing us to exclude them from the baseline models. The discrimination ability (i.e. ability of the model to rank patients' risk) of the baseline models measured via the C-statistic was 0.76 for the TW8 model, and 0.69 for the SVR model. The models provided modest discrimination as assessed by the ROC curves (see Appendix S1).

**Table 1:** Descriptive statistics on potential predictor variables in the total population, and patient used in the TW8 and SVR models, stratified on HCV RNA undetectability.

	Total Population	Used in TW8 Model		Used in SVR Model	
		TW8 HCV-RNA Undetectable	TW8 HCV-RNA Detectable	SVR	No SVR
Total Population	1,111	738	234	696	317
Prior Treatment Experience Type					
Previously Untreated	735	517	155	475	198
Prior partial responder	115	57	46	53	48
Prior relapser	209	164	33	149	49
Prior Null responder	52	Na	na	19	22
IL28B genotype (available on subset)					
C/C	182	167	9	146	26
C/T	346	244	85	235	94
T/T	115	72	37	67	45
HCV-RNA after 4 weeks PR					
Min	24	24	24	24	24
Max	14300000	6480000	14300000	7550000	14300000
Mean	644874	216852	1762829	266375	1347707
Log10 change in HCV RNA from baseline					
Min	−5.96	−5.96	−5.69	−5.96	−5.69
Max	0.44	0.44	0.32	0.44	0.32
Mean	−2.13	−2.65	−0.86	−2.60	−1.20
HCV G1 subtype					
1A	545	352	127	317	174
1B	424	288	79	285	105
Unknown	142	98	28	94	38
Initial Ribavirin dose					
Min	600	600	600	600	600
Max	1400	1400	1400	1400	1400
Median	1200	1200	1200	1200	1200
Age					
Min	21	21	21	21	21
Max	76	74	73	74	76
Mean	51	49.9	51.5	50.1	51.4
Race					
African American	157	74	51	75	62
Non-African American	954	664	183	621	255
Gender					
Male	694	460	149	441	198
Female	417	278	85	225	119
Baseline values (kg)					
Weight					
Min	44.0	44.0	48	45.4	48.0
Max	124.9	124.9	124.7	124.7	124.9
Mean	82.6	83.2	82.1	82.5	83.6
BMI					
Min	17.2	17.2	18.1	17.2	18.1
Max	51.7	51.7	45.7	51.7	47.6
Mean	28.0	28.1	27.7	27.9	28.2
Hemoglobin					
Min	10.4	11.4	10.4	11.4	10.4
Max	19.4	18.7	19.4	18.5	19.4
Mean	14.9	14.9	14.9	14.9	14.9
Steatosis Score					
Unknown	52	28	16	24	24
0	313	223	51	207	77
1	560	379	109	358	150
2	167	99	50	98	57
3	19	9	8	9	9
ALT to ULN ratio					
Min	0.35	0.35	0.35	0.35	0.35
Max	15.65	15.65	7.37	15.65	7.37
Mean	2.00	2.01	2.03	2.07	1.97
Platelets					
Min	49	77	49	77	49
Max	515	515	421	515	481
Mean	239	244.9	215.9	245.5	225.8
HCV RNA Level					
Min	1339	1339	181673	3054	147513
Max	48844754	40176361	48844754	40176361	48844754
Mean	7126734	6855903	8007643	6788434	7833441
Statin use					
Yes	26	22	3	21	4
No	1033	716	231	656	291
Unknown	52	Na	na	19	22

We then performed the same modeling exercise using TW4 decline (log_10_ reduction in plasma HCV RNA from baseline to treatment week 4) in addition to baseline data. When evaluating the candidate predictors individually, IL28B genotype was an important independent predictor at baseline, but was no longer associated with outcomes after adjustment for TW4 decline – TW4 decline had the greatest potential effect on prediction. It has previously been shown in this clinical trial dataset [SPRINT-2, RESPOND-2] that whilst IL28B genotype is an important baseline predictor of outcomes, this effect is attenuated after adjustment for week 4 viral declines.[6] IL28B genotype and prior treatment experience were not selected in the final models because their inclusion did not improve prediction beyond the given model variables. Therefore, we were able to utilize 856 patients for the TW8 model, and 522 patients for the SVR model. The predictive factors in the TW4 model for TW8 response were TW4 decline, race, baseline platelet count and ALT. The predictive factors in the week 4 model for SVR were TW4 decline, G1-subtype, gender, BMI, RBV starting dose and baseline platelet count.

The predictive accuracy of the TW8 and SVR models that included TW4 decline was superior to the baseline models (see Appendix S1). Bootstrapping indicated good validity of the models. The C-statistic of the TW8 model was 0.88 and the SVR model was 0.80. For this reason we developed nomograms only for the TW4 models. The linear predictor used to generate each model is found in Appendix S1.

**Figure 1:**
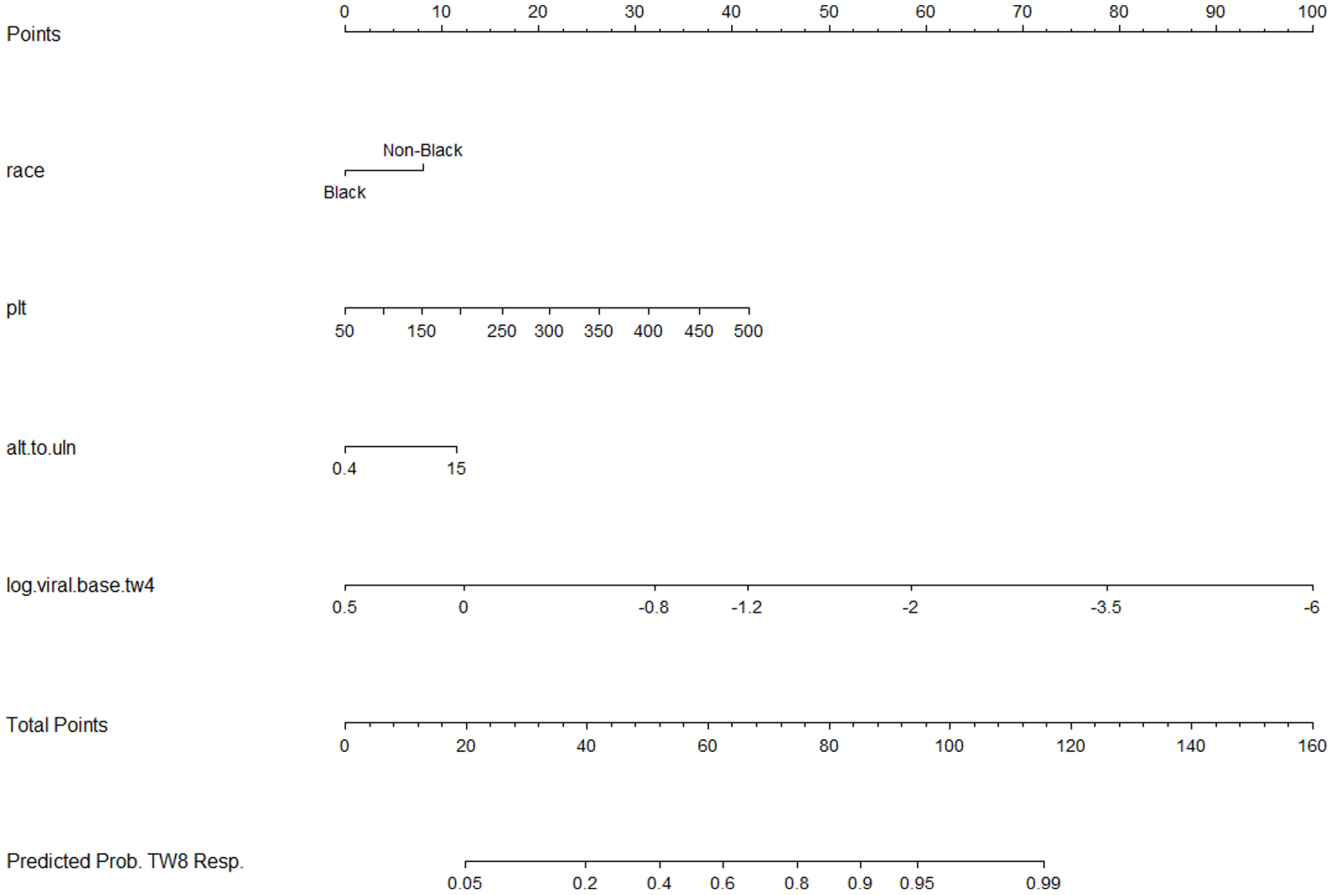
Nomogram for predicting TW8 response in null responders, partial responders, relapsers and previously untreated patients treated with Boceprevir + PR. Instructions: This nomogram is a visual representation of the regression model built to predict TW8 response to boceprevir. It can be used to calculate a patient's predicted probability of becoming undetectable at TW8 if they have initiated boceprevir treatment. To use it, first circle the patients TW4 HCV-RNA on the TW4 HCV-RNA scale. By drawing a straight line upwards to the points scale. This represents the number of points for that patient based upon their TW4 HCV-RNA level. For example, if they have a value of ≤1500, the point score would be 100. Repeat this procedure for each of the variables presented in the nomogram. Once all point scores are determined, sum the total points and circle that value on the Total Points scale after the last variable. Draw a straight line downward from the Total Points scale to determine an individuals predicted probability of a TW8 response.

**Figure 2:**
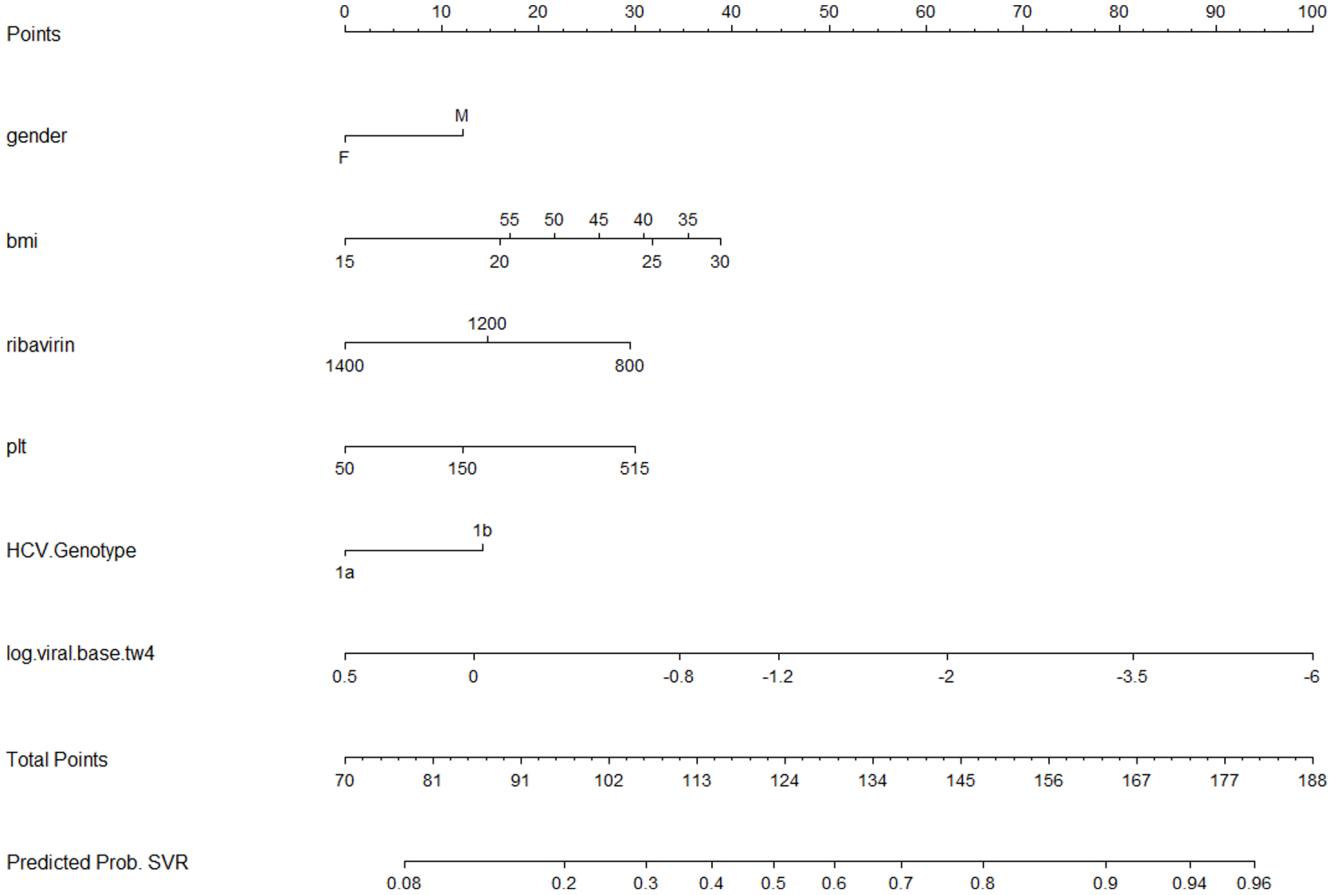
Nomogram for predicting SVR in null responders, partial responders, relapsers and previously untreated patients treated with Boceprevir + PR.

The nomograms for TW8 response and SVR are presented in Figures 1 and 2. Each nomogram can be used to calculate a patient's predicted probability of becoming undetectable at TW8 or achieving SVR if they have initiated and complete boceprevir treatment. To use it, first circle the patient's value for each variable on the individual variables scale. Then, draw a straight line upwards to the points scale. This represents the number of points for a given patient on a given variable. This procedure is repeated for each of the variables presented in the nomogram. Once all point scores are determined, sum the total points and circle that value on the total points scale after the last variable. To determine an individuals predicted probability of a response, draw a straight line downward from the total points scale.

### Decision Trees

The recursive partitioning analyses revealed trees that formed risk groups based on clinical characteristics similar to the nomogram. Patients were categorized in the TW8 tree into six groups based upon the log_10_ reduction in HCV RNA from baseline to treatment week 4 and platelets. Those who had a log_10_ reduction of less than 0.45 had the lowest predicted probabilities, and those with a log_10_ reduction of greater than 1.83 had the highest predicted probabilities. The tree for SVR was more complex, using TW4 decline, HCV-RNA genotype 1 subtype and BMI to create eight groups. Patients who had a TW4 decline of less than 0.7 log_10_ reduction and were HCV-RNA genotype 1a had the lowest overall predicted probabilities, while those who had a log_10_ reduction of greater than 2.36 with a BMI of less than 36 had the highest predicted probabilities. The TW8 and SVR trees had C-statistics of 0.853 and 0.784, respectively, indicating the nomograms provided superior prediction.

### Validation

Finally, we used a dataset from the management of anemia study to externally validate the models/nomograms. The nomograms achieved moderate to excellent predictive ability when tested against the validation dataset. When predicting TW8 response, the nomogram produced excellent results as represented by a C-statistic of 0.85. When predicting SVR, the nomogram produced moderate predictive ability with a C-statistic of 0.71. Calibration curves for both models were assessed and showed good calibration (see Appendix S1).

## Discussion

The development of HCV protease inhibitors represents an important therapeutic advance for patients with genotype 1 HCV. Boceprevir-based triple therapy offers substantial improvements in SVR rates, and many patients will be eligible for shortened duration therapy with 28 vs. 48 weeks.

A number of pre-treatment host and viral factors have been associated with the outcome of BOC triple therapy.[6] These include *IL28B* genotype, race (black vs. non-black), liver fibrosis stage, baseline viral load, HCV-1 subtype (1a vs. 1b), body mass index (BMI), and among treatment-experienced patients, the prior response to PR.[6] In addition to baseline factors, the rate of plasma HCV RNA decline during the early on-treatment period has been identified to be strong negative predictor of outcome. Important thresholds include the failure to achieve a 1-log_10_ drop in plasma HCV RNA at week 4,[6] as well as HCV RNA <1000 IU/mL at week 8,[21] both of which are predict for non-SVR (indeed, the FDA and EMA have recently recognized HCV RNA >1000 IU/mL as a stopping rule for boceprevir). Despite the recognition that these predictors are important, accurate discrimination of likelihood of SVR for an individual patient remains limited. Further, response-guided therapy is an important advance for patient care, and to date there are few data exploring prediction of TW8 response, used as the eligibility criteria for short duration treatment.

We have developed predictive models and corresponding nomograms for predicting TW8 response and SVR in patients initiated on boceprevir plus PR. The models that included only baseline variables were satisfactory, but these analyses were limited by data constraints, in particular the lack of IL28B genotyping data for many patients. Previous studies that developed predictive models for the outcome of PR dual therapy have shown that inclusion of IL28B genotype improves predictive accuracy of baseline models.[22] Further, our data had limited numbers of past treatment failure types, thus limiting our ability to fully explore the impact of past treatment history. However, including TW4 decline in HCV RNA level allowed many of these issues to be overcome. IL28B genotype informs IFN responsiveness, and we have previously shown that TW4 response captures this information.[23] Similarly, prior treatment history informs IFN responsiveness, and this information can be captured in ‘real time’ by using the 4 week PR lead-in.

The more successful models were therefore those that included TW4 decline in plasma HCVRNA level. These TW4 models were better calibrated than those models that included only baseline predictors. The TW4 models performed very well, with high areas under the ROC curve, and c-statistics of 0.88 and 0.80 for TW8 response and SVR, respectively. The models were successfully validated using data from the management of anemia study, which confirmed clinical utility [8], [9].

We used decision tree analysis to compare the nomograms to an approach where patient outcome was assessed according to grouping of identified known prognostic factors. We did this to simulate common risk assessment performed in the clinic. The results were disappointing. Clinicians will achieve greater accuracy using the nomograms over the counting or ad hoc assembly of risk factors through categorized variables.

There are a number of limitations to the current study. One of our stated goals was to develop models using baseline patient characteristics. Unfortunately, the performance characteristics of the baseline models were suboptimal. There were a number of limitations inherent in the datasets available. These analyses were limited by data constraints, in particular the lack of IL28B genotyping data for many patients. Previous studies that developed predictive models for the outcome of PR dual therapy have shown that inclusion of IL28B genotype improves predictive accuracy of baseline models.[22] Further, our data had limited numbers of past treatment failure types, thus limiting our ability to fully explore the impact of past treatment history. Including TW4 decline in HCV RNA level allowed many of these issues to be avoided. IL28B genotype informs IFN responsiveness, and we have previously shown that TW4 response captures this information.[23] Similarly, prior treatment history informs IFN responsiveness, and this information can be captured in ‘real time’ by using the 4 week PR lead-in. Liver fibrosis stage was not included in the final models, despite being known to be associated with the outcome of BOC-based therapy.[6] We made a conscious decision not to include liver fibrosis stage in the model, due to the increasing availability and uptake of non-invasive markers of liver fibrosis stage, however we did assess its potential for inclusion in the models. When we tested the influence of fibrosis stage on the final model, inclusion did not have a great influence on the TW4 models. Finally, the models were externally validated using data from the management of anemia study,[8], [9] which confirmed clinical utility. As is normally the case, the predictive accuracy was not quite as high in the external validation dataset. This is likely to reflect the different characteristics of the cohort enrolled in the management of anemia study, compared to the training cohort: treatment-naïve, non-cirrhotic patients with baseline hemoglobin 13–15g/dL (male) or 12–15g/dL (female).

The data support the clinical utility of the 4-week lead-in period of PR therapy for individualizing therapy, particularly for patients where the decision to treat now or treat later is not clear-cut. This may arise when patients are concerned about tolerability or duration of therapy, or where clinicians are concerned about response rate and the risk of PI resistance, particularly in patients with prior non-response to PR. The 4 week lead-in allows identification of patients who achieve an RVR – these patients will have a high rate of SVR, and addition of boceprevir may not be indicated. The lead-in also allows a period of adjustment to the side effects of treatment before DAA introduction. Importantly, for patients who remain viraemic at week 4, use of these nomograms can now refine prediction of eligibility for short duration therapy, as well as prediction of overall likelihood of success. We believe the nomograms will help maintain patient motivation and compliance throughout therapy. The predicted outcome may also be relevant to a patient's decision to continue with treatment beyond week 4, or to defer PI exposure pending the availability of next generation DAAs.

The data have implications for health care dollar utilization. Currently, the cost for boceprevir is calculated according to the duration of use (cost/week), in contrast to telaprevir that has a fixed 12-week cost. Short duration boceprevir therapy is therefore less expensive than short duration telaprevir therapy. The nomograms identify patients more likely to be eligible for short duration therapy, and for who boceprevir- based therapy may be the more cost effective treatment regimen.

We acknowledge that the treatment landscape is rapidly changing, and the next generation DAAs simeprevir and sofosbuvir have recently been approved by the FDA and the EMA for use in combination with PR. Regimens offer the benefit of once daily dosing, improved tolerability, and increased likelihood of short-duration dosing. In North America, and in the stronger economies of Western Europe, it is likely that these agents will largely supersede boceprevir, limiting the lifespan of the nomogram. However, these new regimens involve a significant price premium. Future IFN-free regimens will be even more expensive. Short duration boceprevir, identified by boceprevir nomograms, may therefore continue to represent a cost-effective regimen for payers in these regions. Moreover, the approval and availability of these next generation DAA regimens in regions with weaker economies is likely to be considerably delayed. The nomograms will continue to be clinically relevant in these regions for an extended period.

**In summary**, we have developed nomograms for predicting response to boceprevir therapy using data from the registration studies. Predictive models incorporating baseline data and TW4 HCV RNA decline provide excellent individual predictions of TW8 response and SVR, and support the clinical utility of the lead-in phase of PR. The nomograms are suitable for point-of-care use to inform individual patient and physician decision-making about the potential duration and success from treatment with boceprevir plus PR.

## Supporting Information

Appendix S1 The following information is available in this file: S1a. Formula of linear predictor for nomogram of TW 8 Response. S1b. Formula of linear predictor for nomogram of sustained virologic response. S1c: Calibration error of TW8 response: baseline variables in partial responders, relapsers and previously untreated patients. S1d: Calibration error of TW8 response: baseline variables plus treatment week 4 HCVRNA levels and log_10_ change in HCV-RNA from baseline to TW4 in null responders, partial responders, relapsers and previously untreated patients. S1e: Calibration error of SVR: baseline variables only in partial responders, relapsers, previously untreated patients. S1f: Calibration error of SVR: baseline variables plus TW4 HCVRNA levels in null responders, partial responders, relapsers and previously untreated patients. S1g: Validation Dataset Predicted Versus Actual Probabilities TW8 Nomogram. S1h: Validation Dataset Predicted Versus Actual Probabilities for SVR Nomogram.
(DOCX)
