# Supplementary material for: Prediction of Treatment Week Eight Response & Sustained Virologic Response in Patients Treated with Boceprevir Plus Peginterferon Alfa and Ribavirin
Source: PLoS One. 2014 Aug 1;9(8):e103370. doi: 10.1371/journal.pone.0103370 (PMC4118864; doi:10.1371/journal.pone.0103370)
Supplement: Appendix S1 — The following information is available in this file: S1a. Formula of linear predictor for nomogram of TW 8 Response. S1b. Formula of linear predictor for nomogram of sustained virologic response. S1c: Calibration error of TW8 response: baseline variables in partial responders, relapsers and previously untreated patients. S1d: Calibration error of TW8 response: baseline variables plus treatment week 4 HCVRNA levels and log10 change in HCV-RNA from baseline to TW4 in null responders, partial responders, relapsers and previously untreated patients. S1e: Calibration error of SVR: baseline variables only in partial responders, relapsers, previously untreated patients. S1f: Calibration error of SVR: baseline variables plus TW4 HCVRNA levels in null responders, partial responders, relapsers and previously untreated patients. S1g: Validation Dataset Predicted Versus Actual Probabilities TW8 Nomogram. S1h: Validation Dataset Predicted Versus Actual Probabilities for SVR Nomogram. (DOCX) [file pone.0103370.s001.docx]

S1a. Formula of linear predictor for nomogram of TW 8 Response

Probability of TW8 = -1.0066084 + 0.63762166 * (race = "Non-Black") + 0.0062064222 * plt + 4.2924814e-08 * max(plt - 156, 0)**3 - 8.4402724e-08 * max(plt - 242, 0)**3 + 4.147791e-08 * max(plt - 331, 0)**3 + 0.37743812 * alt.to.uln - 0.055833207 * max(alt.to.uln - 0.7647, 0)**3 + 0.072974501 * max(alt.to.uln - 1.4697, 0)**3 - 0.017141294 * max(alt.to.uln - 3.76605, 0)**3 - 0.6525488 * log.viral.base.tw4 - 0.04405856 * max(log.viral.base.tw4 + 4.462295, 0)**3 + 0.11578483 * max(log.viral.base.tw4 + 2.001805, 0)**3 - 0.071726268 * max(log.viral.base.tw4 + 0.490425, 0)**3;

S1b. Formula of linear predictor for nomogram of sustained virologic response.

Probability of SVR = -4.9007069 + 0.65603084 * (gender = "M") + 0.17407306 * bmi - 0.0012945072 * max(bmi - 22.5, 0)**3 + 0.0022151291 * max(bmi - 27.4, 0)**3 - 0.00092062194 * max(bmi - 34.29,0)**3 - 1.7435238e-05 * ribavirin - 1.6508718e-08 * max(ribavirin - 800, 0)**3 + 3.3017436e-08 * max(ribavirin - 1000, 0)**3 - 1.6508718e-08 * max(ribavirin - 1200, 0)**3 + 0.0065606481 * plt - 9.5667582e-08 * max(plt - 140.1, 0)**3 + 2.0988534e-07 * max(plt - 238, 0)**3 - 1.1421776e-07 * max(plt - 320,0)**3 + 0.76934476 * (HCV.Genotype = "1b") - 0.39397125 * log.viral.base.tw4 - 0.036515051 * max(log.viral.base.tw4 + 4.346936, 0)**3 + 0.096249836 * max(log.viral.base.tw4 + 1.90605, 0)**3 - 0.059734785 * max(log.viral.base.tw4 + 0.41397, 0)**3;

S1c: Calibration error of TW8 response: baseline variables in partial responders, relapsers and previously untreated patients.
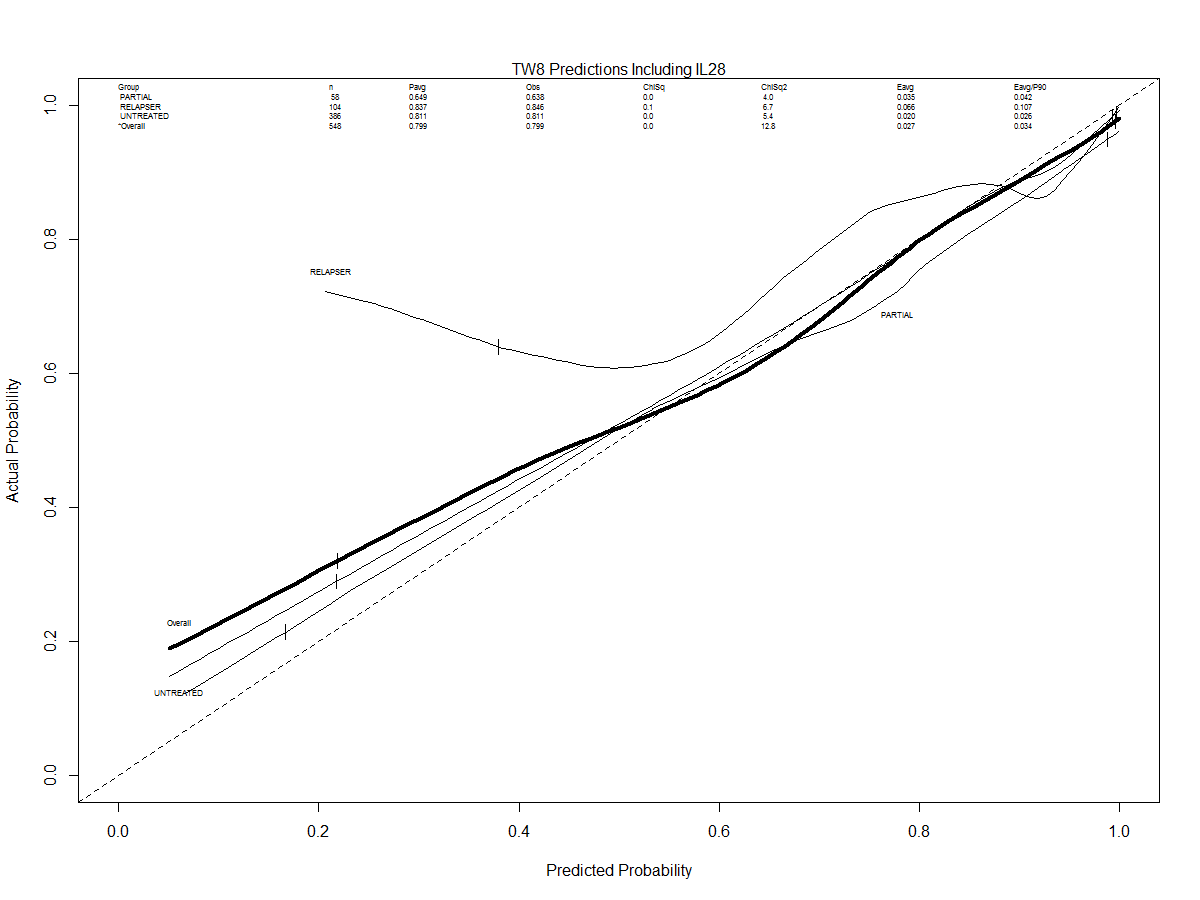


S1d: Calibration error of TW8 response: baseline variables plus treatment week 4 HCVRNA levels and log_10_ change in HCV-RNA from baseline to TW4 in null responders, partial responders, relapsers and previously untreated patients.


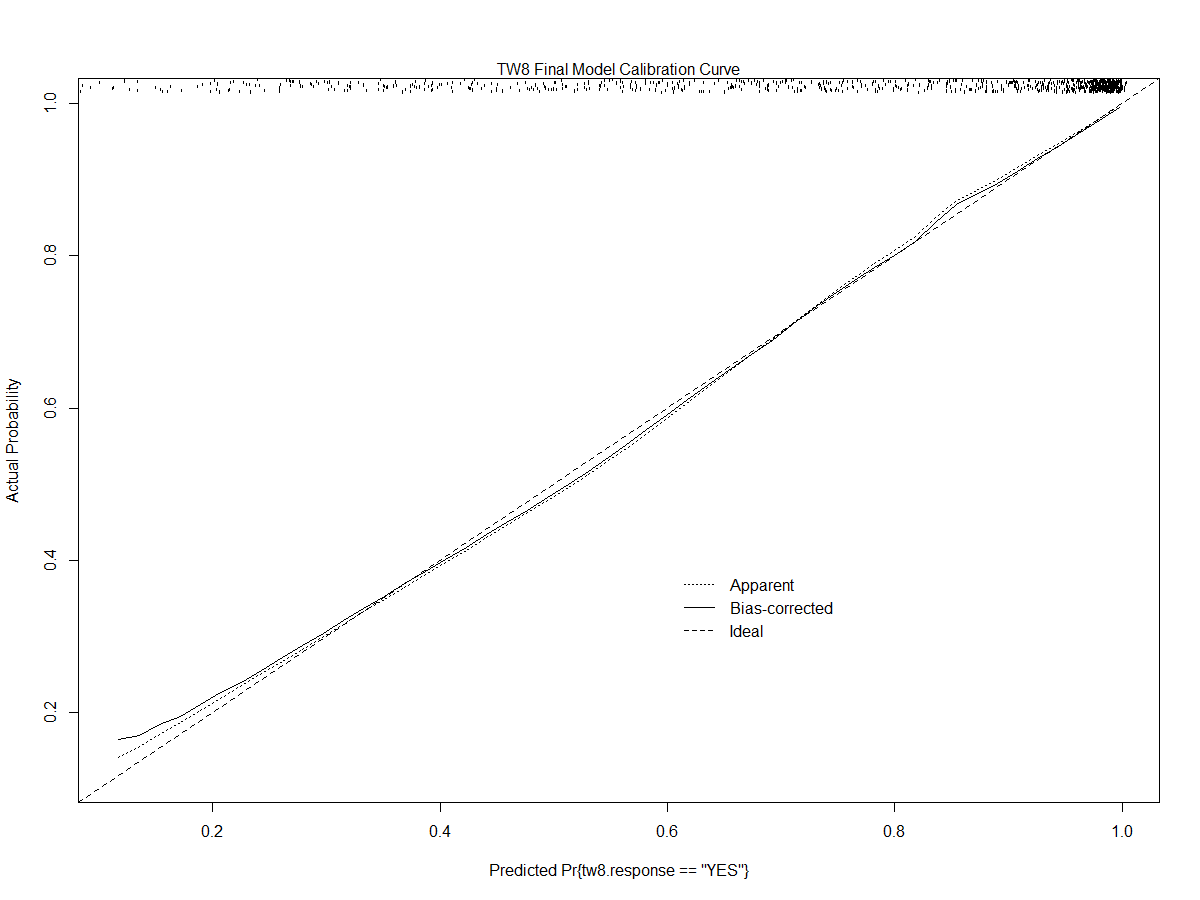


S1e: Calibration error of SVR: baseline variables only in partial responders, relapsers, previously untreated patients.


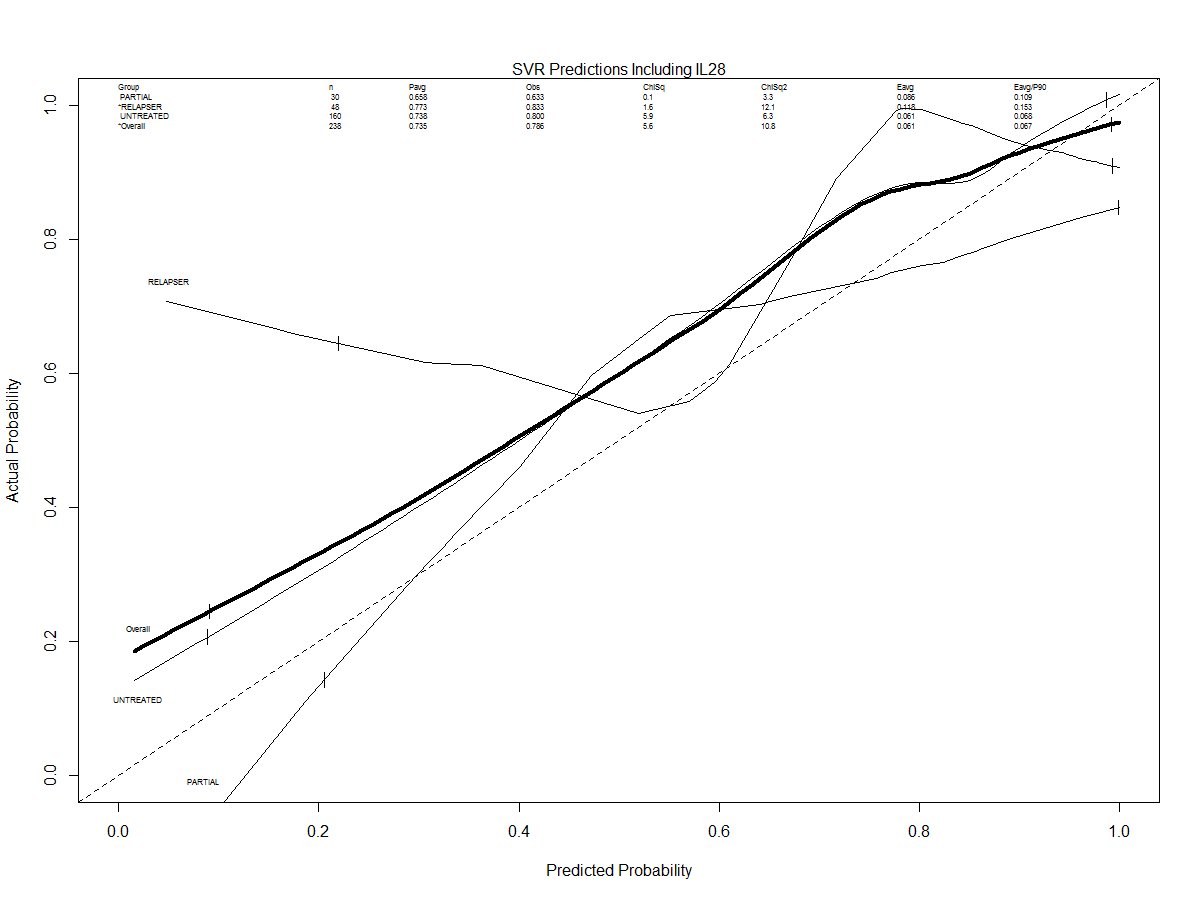


S1f: Calibration error of SVR: baseline variables plus TW4 HCVRNA levels in null responders, partial responders, relapsers and previously untreated patients.


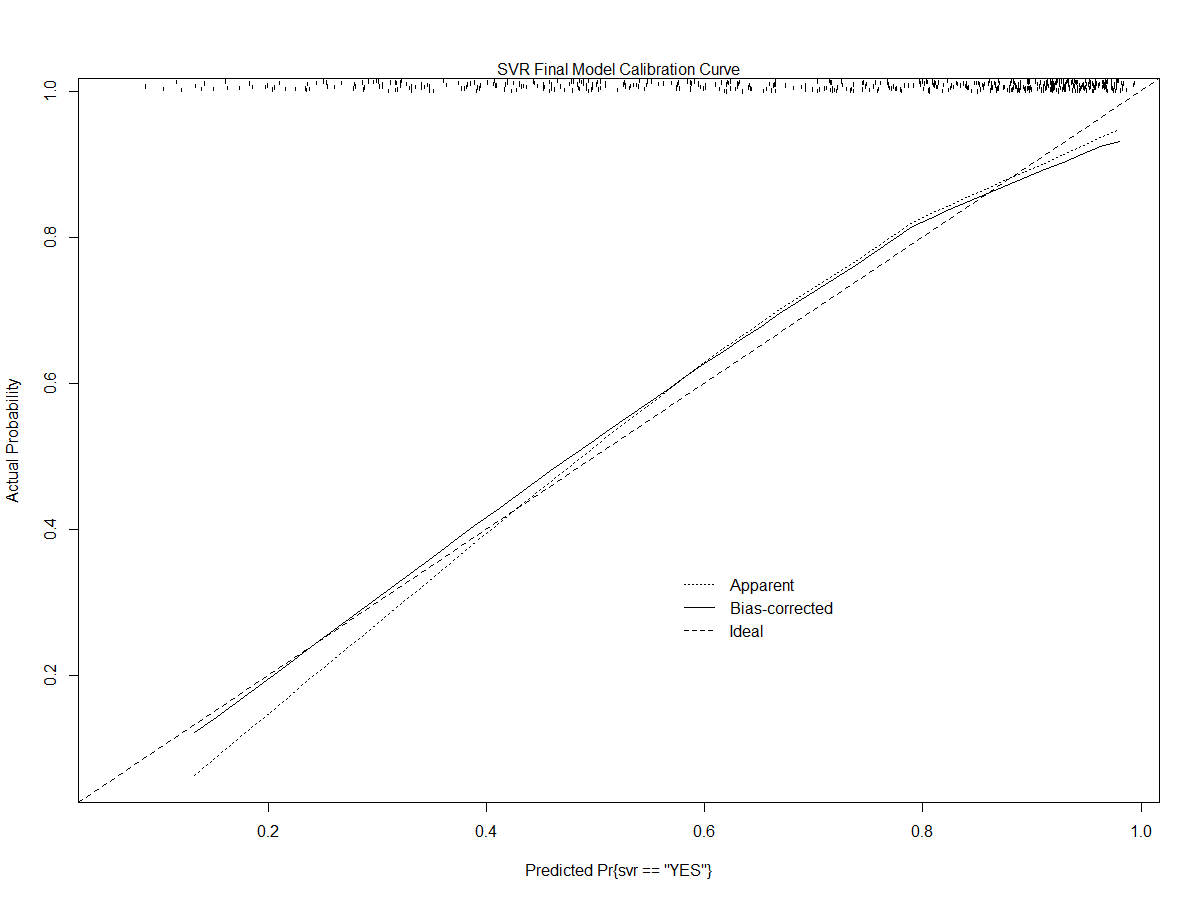


S1g: Validation Dataset Predicted Versus Actual Probabilities TW8 Nomogram





S1h: Validation Dataset Predicted Versus Actual Probabilities for SVR Nomogram
